# Imaging of Kaposi sarcoma

**DOI:** 10.1007/s00261-021-03205-6

**Published:** 2021-07-13

**Authors:** Dhivya Addula, Chandan J. Das, Vikas Kundra

**Affiliations:** 1 University of Texas at Dallas, 800 W Campbell Rd, Richardson, TX 75080 USA; 2 Department of Radiology, All India Institute of Medical Sciences (AIIMS), Ansari Nagar, New Delhi, 110029 India; 3 Department of Abdominal Imaging, The University of Texas MD Anderson Cancer Center, 1400 Pressler St, Houston, TX 77030 USA

**Keywords:** Kaposi’s sarcoma, Imaging, CT, MRI, HIV

## Abstract

Kaposi sarcoma (KS) is a form of cancer that primarily appears on the skin but can potentially involve internal organs. There are several types of KS. The purpose of this article is to discuss the manifestations of KS and their appearance on imaging, the differential diagnoses associated with these findings, and molecular markers associated with KS that can aid appropriate diagnosis and therapy.

## Introduction

Kaposi sarcoma (KS) is a rare angioproliferative malignancy derived from the endothelium discovered by Hungarian dermatologist Moritz Kaposi in the late 1800s [1–3]. Human herpesvirus 8 (HHV8) is the primary cause of KS, but its incidence is also influenced by genetics, the environment, and immunosuppression such as that experienced by patients with acquired immunodeficiency syndrome (AIDS) or immunosuppressive drugs [2, 4]. Although the incidence of KS has been decreasing because of the efficacy of highly active antiretroviral therapy (HAART) for AIDS, KS is associated with patients that have had a solid organ transplant and are immunosuppressed and it is still prominent in Africa, partially due to the difficulty in accessing HAART [5].

In resource-poor areas, diagnosis is often made upon observing the skin lesions, but this has specificity of approximately 80%. Biopsy, including image-guided may be performed to obtain a tissue diagnosis. Histologically, KS is seen as spindle-shaped cells interspersed with abnormal vascular channels [6]. Because of this, it is most commonly confused with vascular or spindle cell tumors; however, KS can be differentiated from these other tumors by the presence of HHV8, detected with the use of molecular methods such as polymerase chain reaction (PCR), direct in situ hybridization, in situ PCR, or reverse transcriptase in situ PCR [7]. HHV-8 (also called KSHV for Kaposi sarcoma-associated herpesvirus) is the causative agent of KS [8] and although it can affect different cell types, endothelial cells are thought to be transformed in KS [9]. HHV8 can cause other diseases and is necessary but not sufficient to cause KS. HHV8 is latent in KS; yet some genes such as LNA-1 (latent nuclear antigen-1), a gene encoding cyclin D homologue (v-cyclin), and a FLIP protein homologue (v-FLIP) are strongly expressed and several signaling pathways are activated including the PI-3 kinase, MAPK, and mTOR pathway; however, the exact mechanism of cellular transformation is still under study.

**Fig. 1:**
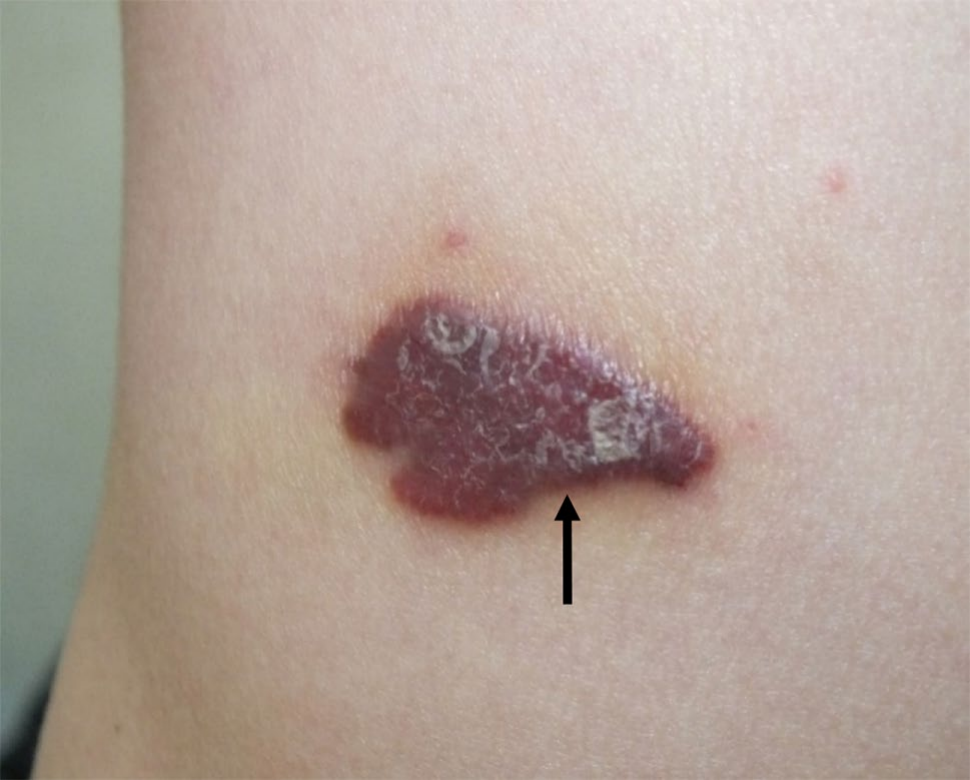
Photograph showing solitary violaceous and dusky erythematous plaque of KS on the anterior trunk. (Picture courtesy of Dr. Neena Khanna, Professor of Dermatology, All India Institute of Medical Sciences (AIIMS), New Delhi, India)

The various types of KS include classic KS, endemic KS, epidemic KS, and iatrogenic immunosuppressed KS [4]. The epidemiology of KS varies for each type; but in general, it is more commonly present in males and can occur in patients of any age [10]. The incidence of KS overall is 481.5 per 100,000 person-years (1.5 in HIV-negative general population, 655.1 in HIV-positive heterosexual men, 172 in women, 1397.1 in HIV-infected men who have sex with men (MSM) among whom it is 180.7 with HAART vs 1271.9 without HAART, 52.9 in children with HIV infection, and 68.6 in transplant recipients per 100,000 person-years). KS frequently occurs in the skin (Fig. 1) and mucosa but can involve the viscera. Among KS types, visceral involvement is more common with the epidemic form and in this type occurs in approximately 15% of cases [11]. Radiologic imaging facilitates the diagnosis, staging, and follow-up of KS. In this article, we discuss the presentation, imaging findings, and management of the various types of KS.

## Kaposi sarcoma variants

KS variants are differentiated primarily by clinical presentation and a radiologist suggesting a potential variant may influence management. The type of variant can affect treatment approach; for example, in addition to other treatments, epidemic KS treatment can include HAART therapy and iatrogenic KS treatment can include reducing immunosuppression or altering the immunosuppressive agent.

### Classic Kaposi sarcoma

The classic variant of KS occurs primarily in older men, typically of Jewish descent, in the Mediterranean and Eastern Europe [5]. It can affect only visceral organs such as the liver, lungs, kidneys, spleen, lymph nodes, or the gastrointestinal (GI) tract without visible skin lesions; but, more commonly does also affect the skin in the lower parts of the body [12, 13]. Skin lesions can present as fungating growths or ulcerated lesions [13]. Treatment of classic KS includes surgery, cryotherapy, radiotherapy, and intralesional and systemic chemotherapy. However, most patients with classic KS have indolent, chronic disease and do not undergo treatment; rather, they undergo surveillance [14].

The staging of classic KS is divided into four categories: In stage one, patients typically have nodules on their legs. In stage two, the lesions take on a plaque morphology primarily affecting large areas of the legs and are locally aggressive, defined as spreading quickly [13, 15]. Stage one and two can be broken down further into Group A, identified by slow disease progression, and Group B, identified by fast disease progression, defined as the quick growth of new or existing skin lesions. Stage three and four are associated with classic KS affecting the viscera and GI tract. In stage three, the lesions are localized to the limbs, whereas in stage four, there are disseminated lesions that also involve the trunk and head. Clinically, by stage three, with visceral involvement, organ damage can occur [13, 15].

### Endemic Kaposi sarcoma

The endemic KS (African KS) variant is most commonly found in Eastern and Central Africa [5]. Four types of endemic KS have been identified, including a benign nodular form, an aggressive localized form, a disseminated form, and a lymphadenopathic form [12]. Male and female children have an equal chance of receiving a diagnosis of endemic KS; but in adults, this variant is more commonly seen in men. It is characterized by small skin lesions in the lower half of the body that slowly become larger. Patients may have symptoms such as itching, burning, and chronic edema [16]. Lymphedema is often seen and can be difficult to control [9].

A pediatric-specific staging classification has been proposed, known as the Lilongwe pediatric KS staging classification. Stage 1 includes those with mild KS, who show lesions on the skin and mouth. Patients classified with Stage 2 present with lymphadenopathic KS that tends to involve lymph node may present with the facial edema, nodular lesions in the mouth, or several lesions across the body among other signs. In Stage 3, known as Woody Edema KS, patients are separated into either 3A (edema < 10% of the body) or 3B (edema > 10% of the body). In Stage 4, patients have visceral or disseminated skin KS [17].

### Epidemic Kaposi sarcoma

The epidemic KS variant, also called HIV-related KS, is most commonly diagnosed in patients who test positive for HIV. Incidence of KS in the setting of HIV is related to the degree of immunosuppression, for example, KS incidence is 384.3 per 100,000 person-years with CD4 count > 200 cells/μL at baseline versus 2050.3 with CD4 count < 200 cells/μL [5]. KS is also one of the AIDS-defining malignancies. After the introduction of highly active antiretroviral therapy (HAART), the number of people in whom epidemic KS was diagnosed decreased significantly [18]. The efficacy of the HAART combination has been due to its lowering HIV replication and to its inhibition of inflammatory cytokines and HIV Tat protein production [19].

Epidemic KS usually presents on the skin, the lining of the mouth, viscera (lungs, GI tract, etc.), or lymph nodes and can appear in more than one location [20]. The course of epidemic KS can differ greatly, with some patients having very limited involvement and others having much more [21]. Differential diagnoses include lymphoma, tuberculosis, Castleman’s disease, and widespread *Mycobacterium avium* complex. Generally, these other conditions are characterized by the appearance of lesions in lymph nodes [22] or viscera with little or no skin involvement. Visceral lesions such as in the lungs and gastrointestinal tract are most commonly seen with Epidemic KS [9].

The staging system of epidemic KS was created by the AIDS Clinical Trials Group (ACTG) and takes into account tumor size, severity of systemic illness related to the HIV virus, and functionality of the patient’s immune system. The staging system is divided into a good risk and poor risk categories based on tumor, immune system, or systemic illness. For the tumor category, good risk is associated with confinement to the skin/lymph nodes, whereas poor risk is associated with visceral involvement. For the immune system category, good risk is associated with a CD4 cell count > 200 per microliter, whereas poor risk is associated with a CD4 cell count < 200 per microliter. For the systemic illness category, good risk is associated with an activity level > 70 on the Karnofsky performance status scale, whereas poor risk is associated with < 70 on the same scale [10]. This staging affects which treatments are chosen. A primary choice is HAART therapy which reduces HIV burden and immunosuppression. It may be used alone or jointly with other treatments, such as radiotherapy, intralesional chemotherapy, cryotherapy, chemotherapy, or interferon-α [18].

It has been suggested that there is a distinct epidemiologic form of KS in men who have sex with men without HIV infection [9]. Skin lesions may occur at any site but tend to be few and visceral or mucosal disease is rare. This usually has an indolent course [9].

### Iatrogenic Kaposi sarcoma

The iatrogenic KS variant can be seen in patients of any age who are immunosuppressed, including those who are taking immunosuppressants after undergoing organ transplantation or for other conditions [5]. Iatrogenic KS generally presents on the skin or in the mucosa, more commonly in lower parts of the body. Visceral involvement is possible in the disseminated form [2]. It is thought that immunosuppression reactivates the HHV8 virus [23]. Treatment for iatrogenic KS includes taking the patient off any immunosuppressant drugs that are not needed; other less common treatments may include wearing elastic stockings, intralesional vincristine, chemotherapy, radiotherapy, silver nitrate cauterization, and surgical excision [2].

## Imaging of Kaposi sarcoma

### Imaging of thoracic Kaposi sarcoma

**Fig. 2:**
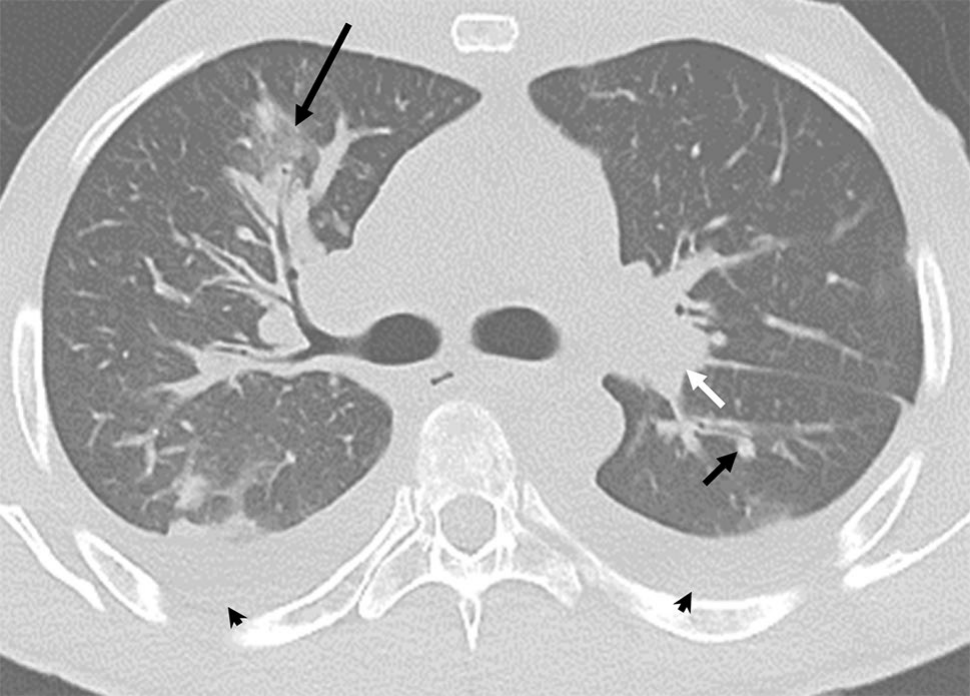
29-year-old man with KS and HIV. Axial CT showing nodular opacities (short arrow) and ground-glass halos (long arrow) surrounding the bronchovascular bundles. Lymphadenopathy (short white arrow) and bilateral pleural effusions (arrowheads) are also noted

In the thorax, KS has the potential to appear in the lungs, pleura, or tracheobronchial tree. On CT scans, the four most common signs of intrathoracic KS include presence of a mass, nodules, thickening of the bronchovascular tree, and pleural effusions (Fig. 2) [24]. Other findings may include ground-glass opacities that may result in halo sign or flame-shaped lesions, round foci, dense interlobular septa, and lymphadenopathy; it is important to consider infection in the differential diagnosis [25]. Among these, pneumocystis pneumonia tends to have perihilar opacities, whereas KS is more commonly associated with infiltrates near blood vessels or near the bronchi [26]. KS pulmonary involvement can result in dyspnea, dry cough, sometimes fever, and hemoptysis that can be life-threatening [9]. Pulmonary Kaposi’s sarcoma in patients with AIDS can portend a short three- to ten-month life-expectancy without effective anti-AIDS therapy [27].

### Imaging of abdominal Kaposi sarcoma

#### Hepatic and splenic Kaposi sarcoma

Hepatic KS is most commonly seen among patients with AIDS, with about 34% of cases diagnosed during autopsy [28]; however, it uncommonly presents during life. Hepatic KS has also been associated with organ transplants [29].

**Fig. 3:**
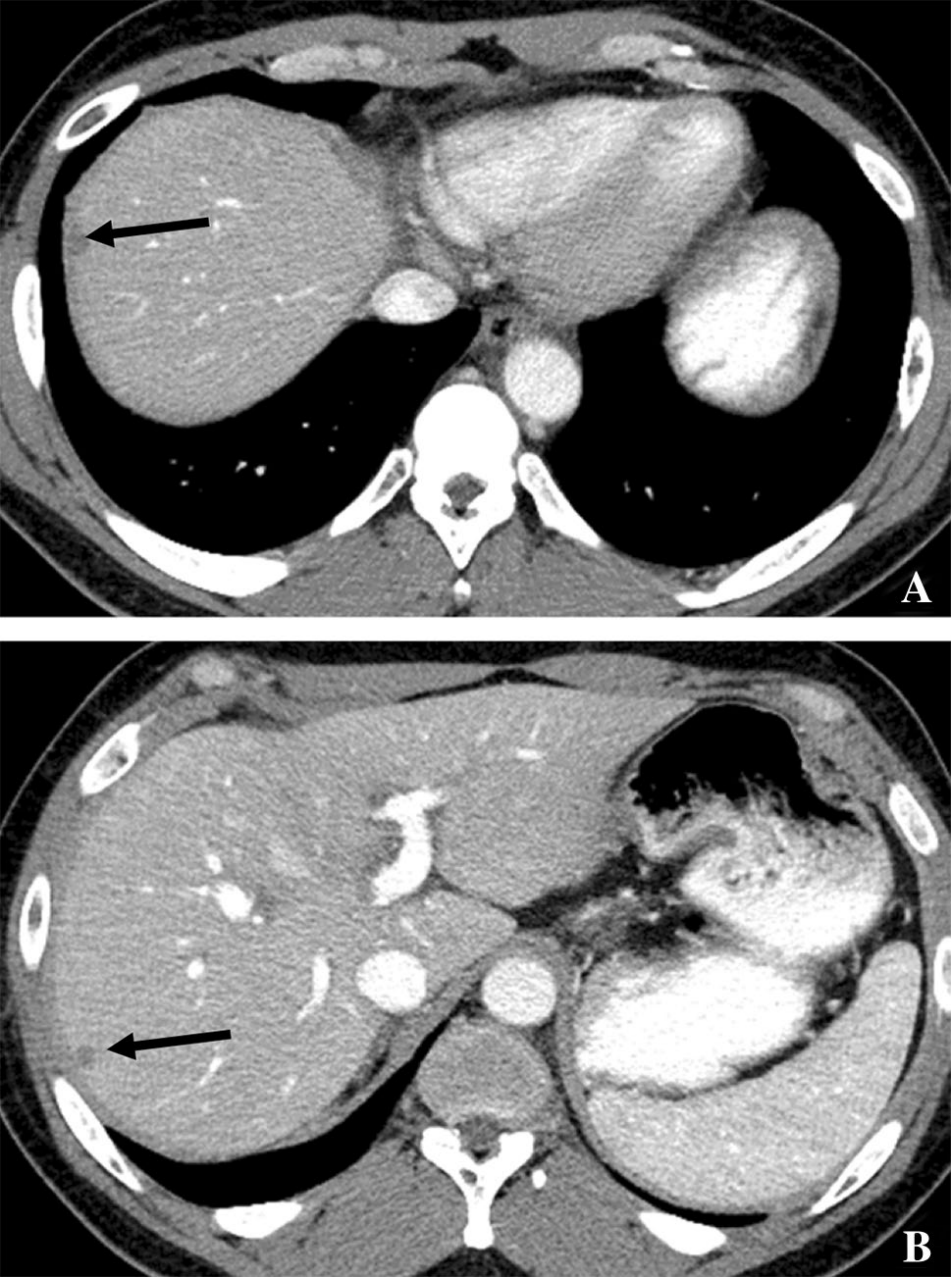
48-year-old man with KS and HIV. Contrast-enhanced axial CT **A**–**B** shows subcapsular low-attenuation lesions in the liver (arrows)

Hepatic KS appears differently on various imaging modalities [30]. A nonspecific finding associated with those who have hepatic KS and AIDS on several modalities is hepatomegaly [30]. KS often appears in the parenchyma near the hilar, portal, or capsular regions of the liver as low-attenuation nodules on CT (Fig. 3) that do not perturb the vessels; [31] but, they can also mimic hemangiomas. On MRI, KS often appears as high-signal nodules on in-phase T1-weighted images and low signal on out-of-phase images presumably due to intracellular fat, isointense on T2, and low signal on 20 min delayed images using a hepatobiliary MR contrast agent [31]. Differential diagnoses include angioma, liver metastases, and fungal microabscess [32]. Splenic KS is extremely rare, and on a microscopic level, it surrounds the arteries of the Malpighian corpuscles and has a stringy appearance. The differential diagnoses include hemangioma, metastasis, and fungal abscess among those who have AIDS [33]. Case reports of CT findings of splenic KS have described splenomegaly [33]. On ultrasound splenic and hepatic KS, lesions usually appear hyperechoic [33]. Histologically, identification of CD31 and CD34 can distinguish hepatic KS from nonvascular spindle cell tumors [28] and identification of HHV8 LNA-1 and D2-40 can help distinguish hepatic KS from vascular tumors [28].

#### Gastrointestinal Kaposi sarcoma

**Fig. 4:**
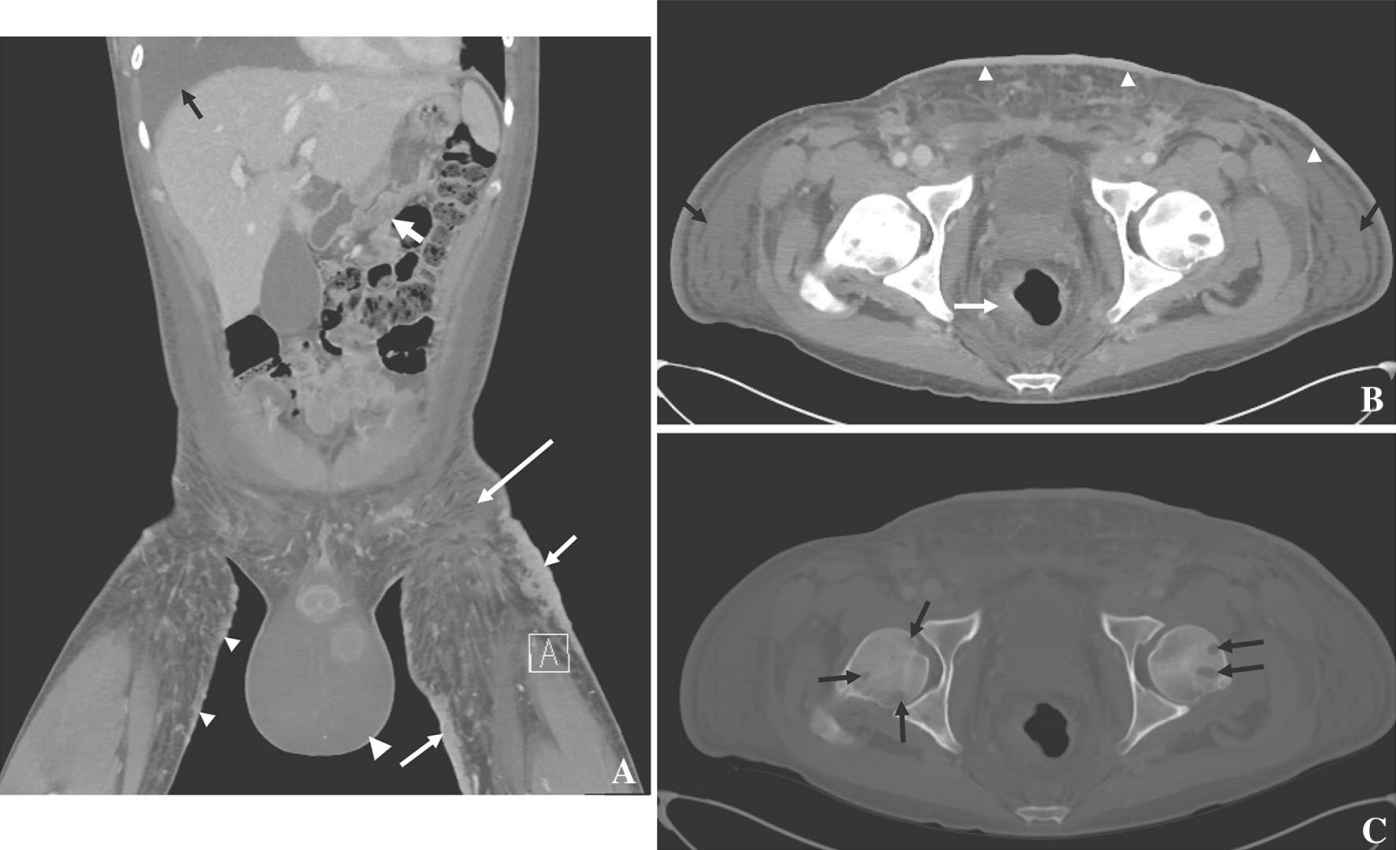
33-year-old male with HIV and KS. **A** Coronal contrast-enhanced CT showing antral wall thickening (fat white arrow), large and thick skin thickening (plaques, short white arrows), small skin nodules (small arrowheads), hydroceles (large arrowhead), subcutaneous edema (long arrow), and a pleural effusion (black arrow). **B** Axial contrast-enhanced CT showing asymmetric rectal wall thickening (white arrow), skin thickening (arrowheads), and subcutaneous edema (black arrows); **C** bone windows show lytic bone metastases

Among untreated AIDS patients, forty to fifty-one percent have visceral involvement of KS, with the gastrointestinal (GI) tract being the most common site [34]. AIDS-related KS more commonly involves the GI tract than the other forms of KS. Any portion of the GI tract may be involved from oropharynx to the rectum. Clinically, KS of the GI tract is most often asymptomatic, only one in five people have symptoms [34–36]. If symptoms are present, they may include hemorrhage, abdominal pain, weight loss, vomiting, diarrhea, and nausea [35, 36]. KS on the skin may suggest GI KS, which is more often noted in the stomach (Fig. 4), duodenum, and biliary tract [34, 37]. GI involvement may be seen as part of more widespread disease such as involving skin, GU system, skeleton, and third spacing of fluids (Fig. 4). Significant complications can include perforation, bleeding, and obstruction [38].

**Fig. 5:**
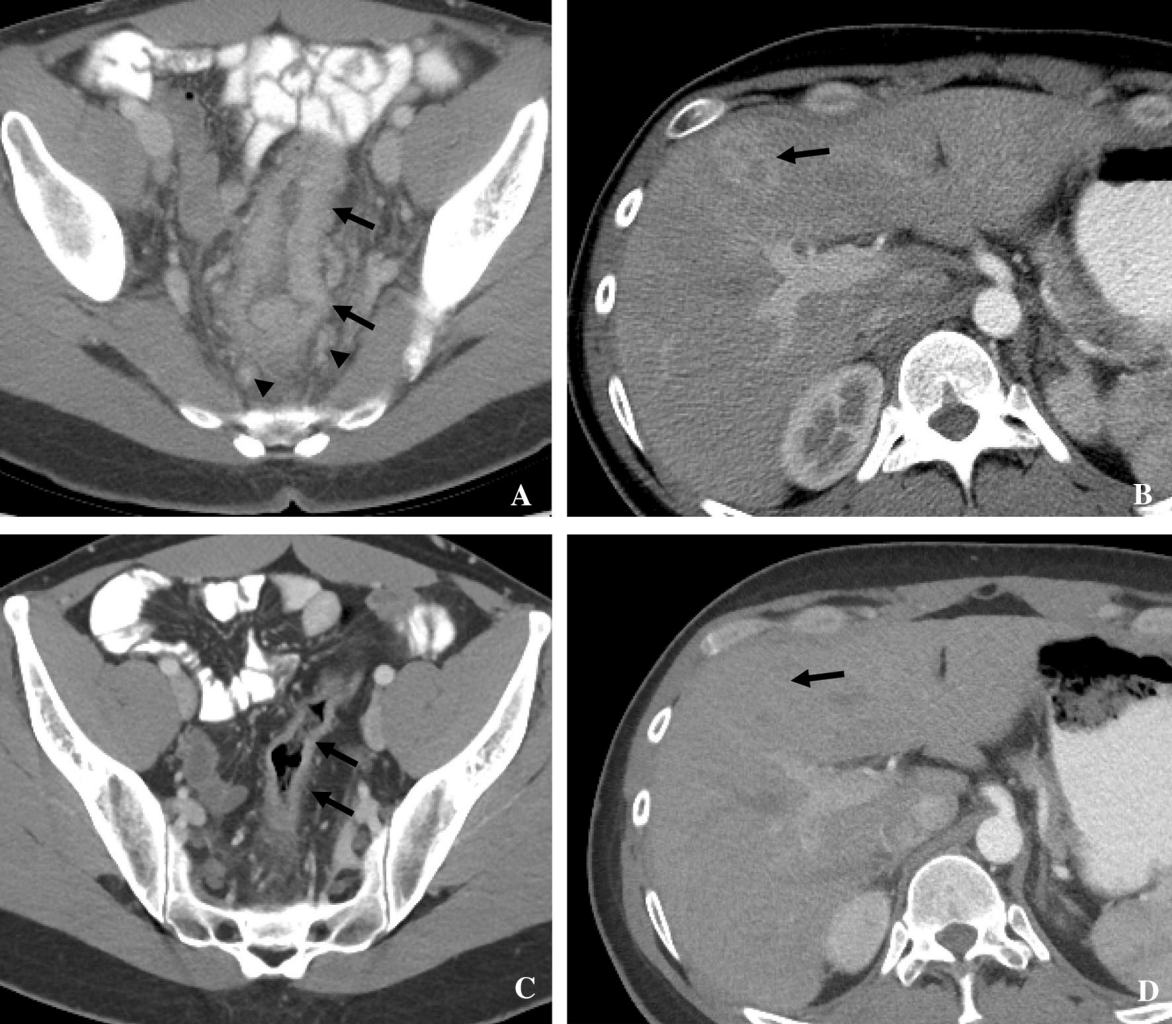
32-year-old male with HIV and KS. Contrast-enhanced CT showing **A** long segment thickening of the recto-sigmoid colon (arrows), enhancing perirectal lymph nodes (arrowheads), and **B** enhancing liver lesion secondary to KS, which resolved post-chemotherapy **C**, **D**

**Fig. 6:**
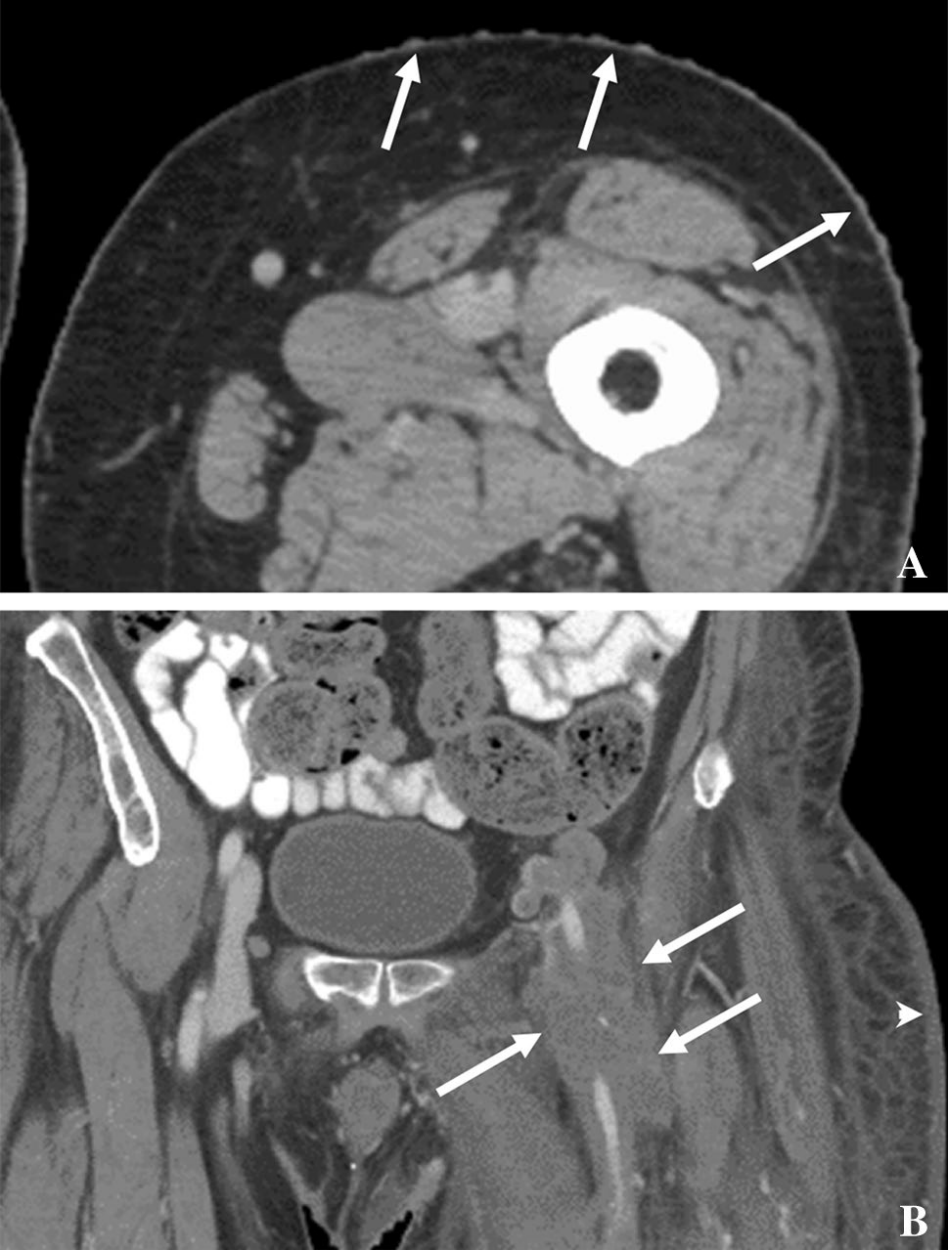
**A** A 56-year-old man with HIV and KS. Contrast-enhanced axial CT of left thigh showing nodular areas of skin thickening consistent with KS (arrows). **B** A 58-year-old man with HIV and KS. Contrast-enhanced coronal CT showing multiple enlarged nodes encasing the left common femoral artery (arrows) consistent with KS. Skin thickening in left gluteal region (arrowhead) is due to skin involvement by KS. Note: These images are of the same patient but from different years

On CT imaging, diffuse or focal wall thickening (Fig. 4) may be seen and there may be associated ascites [34, 39]. Small lesions may present as polypoid or submucosal masses. CT images can also show splenomegaly and often enhancing lymphadenopathy in the mesentery, retroperitoneum, or elsewhere [40]. In gastric KS, particularly, one may see thickened folds, masses, and enhancing lymph nodes [41]. GI KS in the colon can be associated with abdominal cramps and diarrhea [42]. CT scans may show thickening of the colon and lymphadenopathy in the pelvis [42]. KS of the small bowel is uncommon and largely affects patients with HIV [43]. CT scans of a patient with rectal KS may demonstrate wall thickening (Figs. 4, 5), adenopathy, and inflammatory-like changes/fluid adjacent to the rectum [44]. CT can demonstrate improvement after therapy (Fig. 5). Lymphadenopathy can arise along the drainage pathways of visceral disease such as the pelvis and groin in the case of rectal involvement, progress along drainage pathways of skin lesions (Fig. 6), or arise denovo. Differential diagnoses of GI KS include non-Hodgkin lymphoma, leiomyoma, GI stromal tumors, and adenocarcinoma [34, 35] as well as infection, polyps, and inflammatory conditions such as Crohn’s disease. However, uncomplicated KS lesions are less likely to have adjacent fat stranding. Clinical context is important. Because of the typically small tumors, endoscopy can be helpful [34] for detection, characterization, and biopsy for tissue diagnosis. Endoscopically, KS of the GI tract presents as red–purple nodular lesions with little hemorrhage or as macular lesions with more severe hemorrhage [36]. Complications of larger lesions may include bleeding, intestinal or biliary obstruction, intussusception, perforation, diarrhea, and protein-losing enteropathy.

#### Genitourinary Kaposi sarcoma

**Fig. 7:**
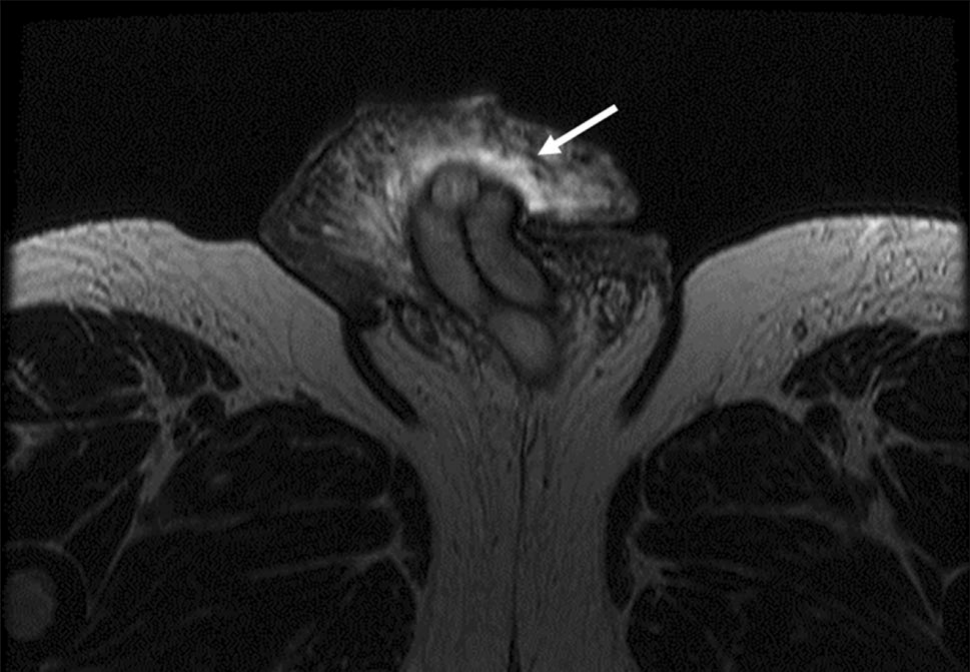
52-year-old man with KS and HIV. MRI of pelvis. T2-weighted axial image showing hyperintense thickened soft tissue in glans penis (arrow)

In men, within the genitourinary tract, KS is more commonly seen on the penis. In women, genital tract involvement is not commonly seen [45]. Lesions along the genital tract such as on the urethral meatus can sometimes lead to outlet obstruction and urinary retention [46]. Imaging studies are rarely needed for evaluation, but can show high T2-signal (Fig. 7), enhancing lesions on MR. KS affecting the scrotum is also extremely rare and very few cases have been recorded [47]; this can include skin involvement and complication such as hydroceles or fluid within the scrotum (Fig. 4). Renal involvement of KS in AIDS patients is not commonly observed at imaging; rather, it is more commonly found during autopsy due to its microscopic nature [48]. As with GI KS, associated lymphadenopathy tends to be hypervascular and manifests as enhancing nodes [49]. Differential considerations include Castleman’s disease, which can co-exist with KS. On contrast CT scans, KS lesions along the GU tract tend to enhance [50]. Differential diagnosis of classic KS on the penis includes pyogenic granuloma, condyloma acuminata, glomus tumor, and angiosarcomas [51]. In addition to antiretroviral therapy in the setting of HIV, radiotherapy, chemotherapy, laser therapy, and surgery represent treatment options [51].

#### Imaging of cutaneous Kaposi’s sarcoma

**Fig. 8:**
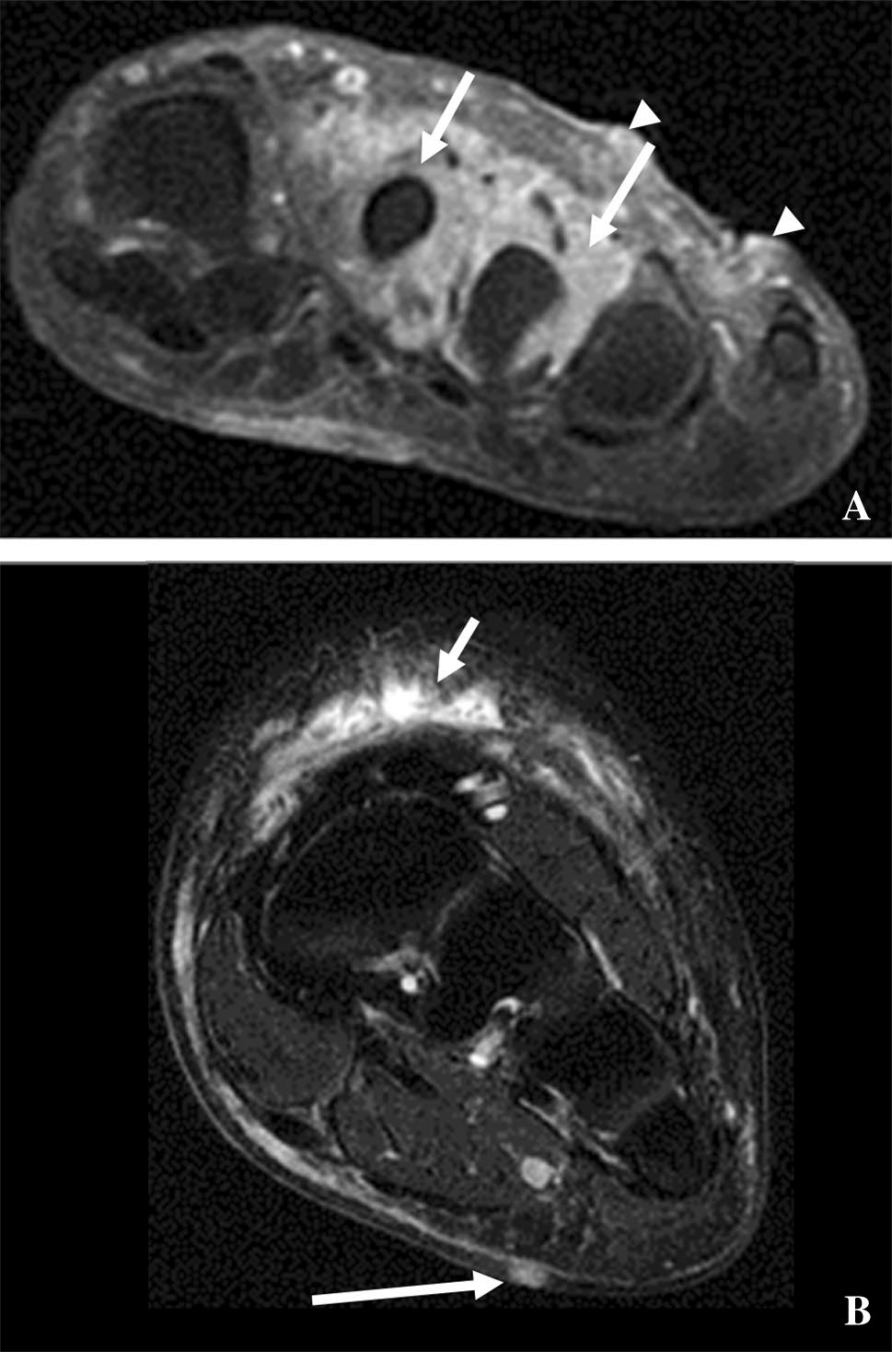
**A** A 67-year-old man with KS. MRI of left foot. Contrast-enhanced, fat-suppressed axial image showing thickened and enhancing soft tissue (arrows) surrounding the second and third metatarsal and enhancing skin nodules (arrowheads). **B** A 45-year-old man with KS. MRI lower extremity T2 fat-saturated axial image with heterogeneously hyperintense subcutaneous tissue (short arrow) and a midline plantar nodule (long arrow), both of which are consistent with KS

KS of the skin can appear virtually on any part of the body such as arms, legs, face, and neck and is normally present in all variants, especially in AIDS-related KS. There is a propensity for involvement of the lower extremities (Figs. 4, 6) and skin thickening may be seen by CT, MR, and ultrasound. Clinically, KS of the skin can appear in nodular, plaque, patch, lymphadenopathic, infiltrative, or florid forms and usually transitions through these phases as the lesions progress. Skin involvement without visceral involvement has a superior prognosis than with visceral involvement; imaging is more commonly performed to evaluate for whether there is visceral involvement (Fig. 4) [52]. MRI will generally allow for a more detailed evaluation of KS involvement [53] with lesions tending to enhance and have increased signal on T2-weighted imaging (Fig. 8).

#### Imaging of Kaposi sarcoma using PET/CT

**Fig. 9:**
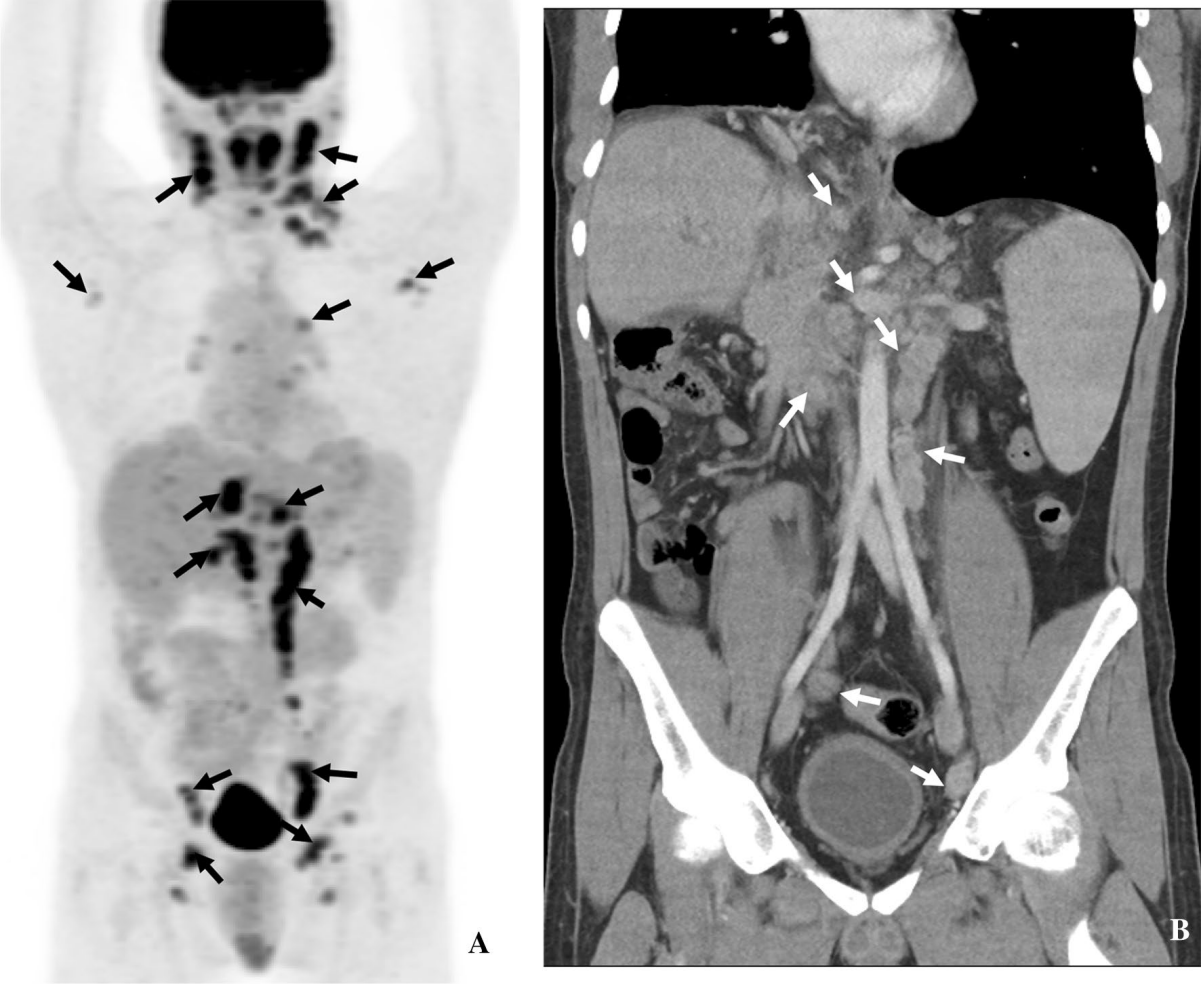
31-year-old male with KS and history of immunosuppression for liver transplant secondary to biliary atresia. **A** PET showing ^18^F-FDG uptake in multi-compartment lymphadenopathy (arrows). **B** Coronal CT showing multicompartment lymphadenopathy in the abdomen and pelvis (arrows)

Using ^18^F-FDG-PET/CT scans can be useful in detecting KS, determining the extent of disease, and assessing which lesions are responding to treatment. On these scans, lesions present with low to moderately increased uptake (Fig. 9) [22] and there can be heterogeneity in uptake among tumors. ^18^F-FDG-PET/CT can be useful in diagnosing and staging cutaneous KS and associated lymph node involvement [54, 55]. Skin uptake can be focal or diffuse and is more commonly seen in the legs and lower torso. In addition, it can help distinguish visceral or skeletal involvement [56], which can portend a worse prognosis. KS is a widespread disease that can be occult and can affect essentially every organ; and ^18^F-FDG-PET/CT scans can be beneficial for whole-body staging [57] (Fig. 9) and evaluating response. In the transplant setting, it can suggest KS, for example with multi-lymph node involvement (Fig. 9), even in the uncommon circumstance of no cutaneous KS lesion [55].

#### Molecular markers

Molecular markers help distinguish KS from other diseases. Histologically, HHV8 expresses LANA-1, and its presence in KS cells indicates the presence of the virus. In addition, expression of viral interleukin-6 (vIL-6) by HHV8 has been associated with progression of KS. LANA-1 is present in more than 90% of patients with KS [7]. For classic KS, in particular, high levels of plasma markers CXCL10 (chemokine), sIL-1RII, sIL-2RA (protein), and CCL3 (chemokine) are associated with an increased risk of KS [58].

### Relationship of Kaposi sarcoma and HIV

**Fig. 10:**
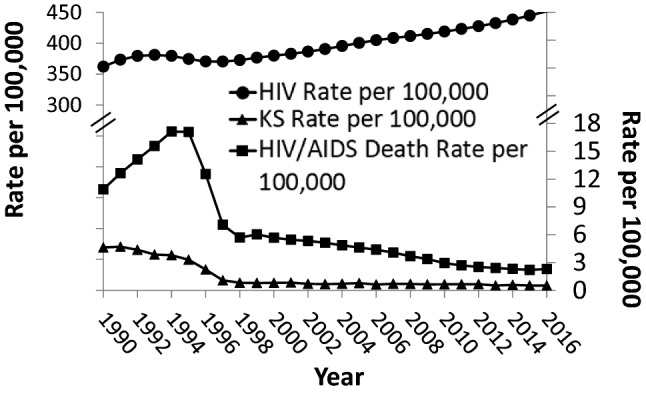
KS rate per 100,000 based on the SEER 9 Areas (San Francisco, Connecticut, Detroit, Hawaii, Iowa, New Mexico, Seattle, Utah, and Atlanta) age-adjusted to the 2000 US Standard Population in relation to the HIV rate per 100,000 and the HIV/AIDS death rate per 100,000 in the USA [60–62]

The incidence of KS increased with the start of HIV infections in humans and the AIDS epidemic. Antiretroviral drugs to treat HIV were discovered starting in the late 1980s and eventually led to the combination HAART treatment. In 1987, azidothymidine (AZT) was made available to the public, and in 1991, nucleoside reverse transcriptase inhibitors were introduced [59]. In 1995, protease inhibitors, such as ritonavir, were released [59, 60] and very likely contributed to the decline in KS incidence seen before 1996 (Fig. 10). After the combination HAART became available in 1996, the incidence of KS in patients with HIV decreased drastically due to effective treatment (Fig. 10) and in part due to AIDS prevention efforts; however, KS can still be found in patients with uncontrolled HIV infection or AIDS today and is still found in non-AIDS-related types of KS [61, 62]. Of note, although the incidence of AIDS and KS has significantly decreased, HIV infection rates were still increasing as of 2016.

## Conclusion

KS is no longer as prevalent as it once was due to the successful introduction of HAART for AIDS. However, it is still found in such patients and in other KS variants such as in iatrogenic immunosuppressed patients, for example related to organ transplants, as well as in classic and endemic forms. It can present with a myriad of manifestations reflected upon imaging of the abdomen and pelvis in addition to other parts of the body. Imaging using modalities such as CT, MRI, and PET/CT scans can aid in diagnosing, staging, and follow-up of KS.

## References

[1] Oriel JD (1997). *Moritz Kaposi (1837*-*1902)*. Int J STD AIDS.

[2] Brambilla L, Tourlaki A, Genovese G (2017). *Iatrogenic Kaposi’s Sarcoma: a Retrospective Cohort Study in an Italian Tertiary Care Centre*. Clin Oncol (R Coll Radiol).

[3] Kasturia SE (2017). *Severe Kaposi Sarcoma in an Urban Public Hospital*. AIDS Res Hum Retroviruses.

[4] Kamyab K (2014). *Demographic and histopathologic study of Kaposi’s sarcoma in a dermatology clinic in the years of 2006 to 2011*. Acta Med Iran.

[5] Liu Z (2018). *The world*-*wide incidence of Kaposi’s sarcoma in the HIV/AIDS era*. HIV Med.

[6] Stein ME (1994). *Radiation therapy for non*-*AIDS associated (classic and endemic African) and epidemic Kaposi’s sarcoma*. Int J Radiat Oncol Biol Phys.

[7] Wang X (2010). *Classic Kaposi’s sarcoma in Han Chinese and useful tools for differential diagnosis*. Oral Oncol.

[8] Dupin N (2020). *Update on oncogenesis and therapy for Kaposi sarcoma*. Curr Opin Oncol.

[9] Cesarman E (2019). Kaposi sarcoma. Nat Rev Dis Primers.

[10] Tappero JW (1993). *Kaposi’s sarcoma. Epidemiology, pathogenesis, histology, clinical spectrum, staging criteria and therapy*. J Am Acad Dermatol.

[11] Bower M (2014). *Prospective stage*-*stratified approach to AIDS*-*related Kaposi’s sarcoma*. J Clin Oncol.

[12] Friedman-Kien AE, Saltzman BR (1990). *Clinical manifestations of classical, endemic African, and epidemic AIDS*-*associated Kaposi’s sarcoma*. J Am Acad Dermatol.

[13] Ramirez K (2016). *Classic Kaposi’s sarcoma* - *complete response to radiation therapy: a case report*. J Med Case Rep.

[14] Nakajima T (2016). *Case of classic Kaposi’s sarcoma*. J Dermatol.

[15] Brambilla L (2003). *Staging of classic Kaposi’s sarcoma: a useful tool for therapeutic choices*. Eur J Dermatol.

[16] Ziegler JL (1993). *Endemic Kaposi’s sarcoma in Africa and local volcanic soils*. Lancet.

[17] El-Mallawany NK (2018). *Proposal of a Risk*-*Stratification Platform to Address Distinct Clinical Features of Pediatric Kaposi Sarcoma in Lilongwe*. Malawi. J Glob Oncol.

[18] Aldenhoven M, Barlo NP, Sanders CJ (2006). *Therapeutic strategies for epidemic Kaposi’s sarcoma*. Int J STD AIDS.

[19] Nasti G (2003). *AIDS*-*related Kaposi’s Sarcoma: evaluation of potential new prognostic factors and assessment of the AIDS Clinical Trial Group Staging System in the Haart Era*–*the Italian Cooperative Group on AIDS and Tumors and the Italian Cohort of Patients Naive From Antiretrovirals*. J Clin Oncol.

[20] Chachoua A (1989). *Prognostic factors and staging classification of patients with epidemic Kaposi’s sarcoma*. J Clin Oncol.

[21] Dezube BJ (2000). *Acquired immunodeficiency syndrome*-*related Kaposi’s sarcoma: clinical features, staging, and treatment*. Semin Oncol.

[22] Morooka M (2010). *Whole*-*body 18F*-*fluorodeoxyglucose positron emission tomography/computed tomography images before and after chemotherapy for Kaposi sarcoma and highly active antiretrovirus therapy*. Jpn J Radiol.

[23] Lebbe C, Legendre C, Frances C (2008). *Kaposi sarcoma in transplantation*. Transplant Rev (Orlando).

[24] Khalil AM (1995). *Intrathoracic Kaposi’s sarcoma*. CT findings. Chest.

[25] Zeschnigk T (1997). *CT and supplementary HR*-*CT of Kaposi’s sarcoma of the pulmonary parenchyma*–*the morphology of the findings and the diagnostic value*. Rofo.

[26] Pozniak AL (1992). *Pulmonary Kaposi’s sarcoma in Africa*. Thorax.

[27] Peer FI (2008). *99mTc*-*MIBI imaging of AIDS*-*related Kaposi’s sarcoma in the lungs*. Nucl Med Commun.

[28] Thampy R (2017). *Imaging features of rare mesenychmal liver tumours: beyond haemangiomas*. Br J Radiol.

[29] Dollard SC (2018). *Donor*-*derived Kaposi’s sarcoma in a liver*-*kidney transplant recipient*. Am J Transplant.

[30] Van Leer-Greenberg B, Kole A, Chawla S (2017). *Hepatic Kaposi sarcoma: A case report and review of the literature*. World J Hepatol.

[31] Tacconi D (2012). *Hepatic Kaposi’s sarcoma in a patient affected by AIDS: Correlation between histology and imaging*. J Ultrasound.

[32] Luburich P (1990). *Hepatic Kaposi sarcoma in AIDS: US and CT findings*. Radiology.

[33] Valls C (1991). *Hepatosplenic AIDS*-*related Kaposi’s sarcoma*. Gastrointest Radiol.

[34] Lee AJ (2015). *Gastrointestinal Kaposi’s sarcoma: Case report and review of the literature*. World J Gastrointest Pharmacol Ther.

[35] Arora M, Goldberg EM (2010). *Kaposi sarcoma involving the gastrointestinal tract*. Gastroenterol Hepatol (N Y).

[36] Friedman SL, Wright TL, Altman DF (1985). *Gastrointestinal Kaposi’s sarcoma in patients with acquired immunodeficiency syndrome*. Endoscopic and autopsy findings. Gastroenterology.

[37] Nidimusili AJ, Eisa N, Shaheen K (2013). *Gastrointestinal Kaposi’s Sarcoma Presenting as Ileocolic Intussusception*. N Am J Med Sci.

[38] Hauser N (2015). *Visceral Kaposi’s Sarcoma Presenting as Upper Gastrointestinal Bleeding*. Case Rep Gastrointest Med.

[39] Gonzalez-Moreno EI (2015). *Gastrointestinal Kaposi’s sarcoma involving stomach, duodenum, and colon*. Ann Gastroenterol.

[40] Leibman AJ, Gold BM (1986). *Gastric manifestations of autoimmune deficiency syndrome*-*related Kaposi’s sarcoma on computed tomography*. J Comput Tomogr.

[41] Virmani V (2012). *Neoplastic stomach lesions and their mimickers: spectrum of imaging manifestations*. Cancer Imaging.

[42] Olanipekun T (2019). *Lower Gastrointestinal Kaposi Sarcoma in HIV/AIDS: A Diagnostic Challenge*. Gastrointest Tumors.

[43] Halankar J, Martinovic E, Hamilton P (2015). *Kaposi’s Sarcoma Presenting as Acute Small Bowel Obstruction Diagnosed on Multidetector Computed Tomography with Histopathological Correlation*. Case Rep Radiol.

[44] Kumar A, Nautsch D (2016). *Kaposi’s Sarcoma of the Rectum in a Homosexual Male with HIV*-*AIDS*. ACG Case Rep J.

[45] Barroso Dos Reis HL (2019). *Genital Kaposi sarcoma in a HIV and syphilis co*-*infected patient: case presentation.*. BMC Infect Dis.

[46] Pantanowitz L, Dezube BJ (2008). *Kaposi sarcoma in unusual locations*. BMC Cancer.

[47] Yenice MG (2018). *Scrotal Kaposi’s Sarcoma in HIV*-*negative patient: A case report and review of the literature*. Turk J Urol.

[48] Gore RM, Miller FH, Yaghmai V (1998). *Acquired immunodeficiency syndrome (AIDS) of the abdominal organs: imaging features*. Semin Ultrasound CT MR.

[49] Nair V (2019). *An unusual case of Kaposi sarcoma masquerading as cystitis in a kidney transplant recipient*. Transpl Infect Dis.

[50] Rha SE, Byun FH, Kim HH, Baek JH, Hwang TK, Kang SJ (1998). *Kaposi’s sarcoma involving a transplanted kidney, ureter and urinary bladder: ultrasound and CT findings*. Br J Radiol.

[51] Kuriyama Y (2018). *Case of classic Kaposi sarcoma of the penis successfully treated with radiotherapy*. J Dermatol.

[52] Beatrous SV (2017). *Cutaneous HIV*. Dermatol Online J.

[53] Guan CS (2018). *MRI findings of AIDS*-*related giant facial Kaposi’s sarcoma: A case report*. Medicine (Baltimore).

[54] Davison JM (2011). *FDG PET/CT in patients with HIV*. AJR Am J Roentgenol.

[55] Reuter S (2014). *A challenging case of rapid progressive Kaposi sarcoma after renal transplantation: diagnostics by FDG PET/CT*. Medicine (Baltimore).

[56] Cengiz A (2016). *18F*-*Fluorodeoxyglucose Positron Emission Tomography/Computed Tomography Imaging in a Patient with HIV (*-*) Kaposi Sarcoma*. Mol Imaging Radionucl Ther.

[57] Liu Y (2011). *Demonstrations of AIDS*-*associated malignancies and infections at FDG PET*-*CT*. Ann Nucl Med.

[58] Aka PV (2015). *A multiplex panel of plasma markers of immunity and inflammation in classical kaposi sarcoma*. J Infect Dis.

[59] Nunes AA (2015). *Profile analysis of patients with HIV/AIDS hospitalized after the introduction of antiretroviral therapy*. Cien Saude Colet.

[60] Markowitz M (1995). *A preliminary study of ritonavir, an inhibitor of HIV*-*1 protease, to treat HIV*-*1 infection*. N Engl J Med.

[61] Engels EA (2008). *Cancer risk in people infected with human immunodeficiency virus in the United States*. Int J Cancer.

[62] Robbins HA (2014). *Epidemiologic contributions to recent cancer trends among HIV*-*infected people in the United States*. AIDS.

